# A Global Bibliometric Analysis on Antibiotic-Resistant Active Pulmonary Tuberculosis over the Last 25 Years (1996–2020)

**DOI:** 10.3390/antibiotics11081012

**Published:** 2022-07-27

**Authors:** Md Asiful Islam, Shoumik Kundu, Tengku Muhammad Hanis, Khalid Hajissa, Kamarul Imran Musa

**Affiliations:** 1 Department of Haematology, School of Medical Sciences, Universiti Sains Malaysia, Kubang Kerian 16150, Kelantan, Malaysia; 2 Institute of Metabolism and Systems Research, University of Birmingham, Birmingham B15 2TT, UK; 3 Department of Biochemistry and Molecular Biology, Jahangirnagar University, Savar, Dhaka 1342, Bangladesh; shoumikk33@gmail.com; 4 Department of Community Medicine, School of Medical Sciences, Universiti Sains Malaysia, Kubang Kerian 16150, Kelantan, Malaysia; tengkuhanismokhtar@gmail.com; 5 Department of Medical Microbiology & Parasitology, School of Medical Sciences, Universiti Sains Malaysia, Kubang Kerian 16150, Kelantan, Malaysia; khalidhaj@usm.my; 6 Department of Zoology, Faculty of Science and Technology, Omdurman Islamic University, P.O. Box 382, Omdurman 14415, Sudan

**Keywords:** tuberculosis, antibiotic, drug, resistance, bibliometrics

## Abstract

Background: Tuberculosis (TB) is still a leading global cause of mortality and an increasingly crucial problem in fighting TB is antibiotic resistance. We aimed to conduct a bibliometric analysis on the articles of the past 25 years on antibiotic-resistant active pulmonary TB. Methods: Appropriate keywords were combined using the Boolean and wildcard operators and searched in Scopus database for articles published between 1996 and 2020 in English language. For all the bibliometric analyses, the *Bibliometrix* package in RStudio and *Biblioshiny* web apps were used. We identified the publication and citation trends, topmost cited documents, most productive authors, countries and institutions and most influential journals and funding agencies. We constructed collaborative networks of countries and co-citations. In addition, we developed a Three-Fields plot and a Thematic Map to explore different publication themes. Results: We included 7024 articles (88.9% research articles) and a persistently increasing publication and citation trends were evident throughout the past 25 years. Boehme 2010 was the most cited paper (1609 times cited), Stefan Niemann was the most productive author (86 papers), and ‘International Journal of Tuberculosis and Lung Disease’ was the leading journal. Centers for Disease Control and Prevention was the top contributing institution (3.7% papers) and both US- and UK-based funders were leading. The most productive countries were the USA, India, the UK, South Africa, and China and most of the collaborations took place between the USA, the UK, and South Africa. Conclusion: Undoubtedly, researchers and funders from the USA dominated followed by the UK in most of the fields in antibiotic-resistant TB research. The outcomes of antibiotic-resistant TB research would be more productive and translational if researchers from low- or middle-income countries (especially from Africa, South America and Asia) with high research productivity and TB burden could be in collaboration with high-income countries exhibiting low TB burden.

## 1. Introduction

Tuberculosis (TB) is one of the leading infectious diseases caused by *Mycobacterium tuberculosis*, accounting for almost one-ninth of the total deaths worldwide. To reduce incidence, mortality, and end the global TB epidemic, long-term plans, such as the Sustainable Development Goals and the End TB Strategy, are being carried on with the similar mission of reducing the death rate to 90% and incident rate to 80% in 2030 compared to that of 2015 [1]. Global epidemiological data illustrates a continuous decline in TB incidence and TB-associated deaths in the recent years, however, resistance to antibiotics - that are being used for treating TB patients is turning out to be an obstacle to achieve the expected outcomes [2,3]. Some recent meta-analyses illustrate that about 42.6–47.0% of TB patients are resistant to any of the first-line anti-TB drugs. Moreover, the increase of multidrug-resistant TB (MDR-TB) cases (TB patients resistant to both isoniazid and rifampicin) and extensive drug-resistant TB cases (MDR-TB patients who are also resistant to any of the fluoroquinolones and at least any of the three injectable second-line drugs namely amikacin, kanamycin and capreomycin) and total drug-resistant TB cases (TB patients resistant to all first- and second-line anti-TB drugs) are threatening the appropriate management and treatment of TB patients [4,5,6]. In 2019, drug-resistant TB accounted for the death of almost half a million people worldwide and 78% of the rifampicin-resistant TB patients developed MDR-TB. As the cases of TB are not uniformly distributed across the world, it is difficult for the high-burden TB countries to ensure proper management, treatment and care of TB patients because those countries are generally low- and middle-income countries and people are mostly homeless, under- or malnourished, smokers, or HIV-infected and each of these conditions serve as a risk factor for TB along with drug resistance [1,7]. 

Bibliometric analysis is a tool that involves the use of mathematical and statistical methods to measure the growth, productivity and overall trend of publications on a particular issue. Bibliometric analyses in the medical field are essential to demonstrate the trend and changes in research and consequently, it encourages researchers to scrutinize which countries and institutions are advancing and which fields are lagging and how to improve [8,9]. A previous bibliometric analysis (2007–2016) associated with TB [10] represented the overall scenario of publications by analysing the frequency of publications in different countries, regions, times, journals and citations. Therefore, the objective of this bibliometric analysis was to understand the trend and progression of global research outcomes comprehensively on antibiotic-resistant active pulmonary TB in the past 25 years (1996–2020).

## 2. Results

### 2.1. Search Results

**Figure 1:**
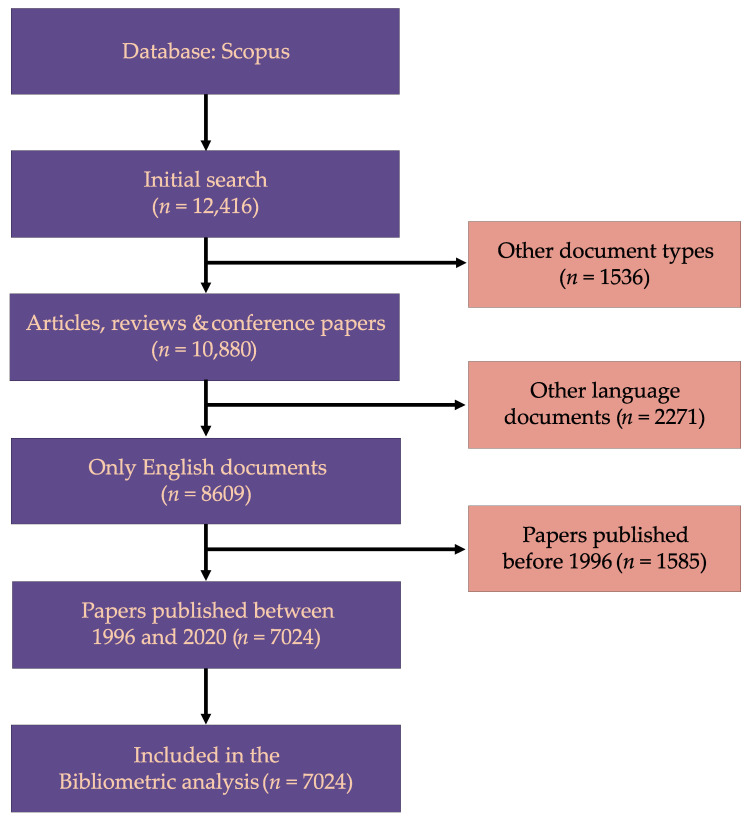
Flow diagram of study selection for the bibliometric analysis.

From the Scopus database search, a total of 12,416 papers were initially found and based on the eligibility criteria, after excluding 5392 ineligible studies, 7024 papers were included in this bibliometric analysis (Figure 1).

### 2.2. Major Characteristics of the Included Studies

Among the included studies, the majority of the papers were of original articles (88.4%), followed by review articles (9.7%) and conference papers (1.9%) with an average citation per document and per document per year were 28.2 and 2.8, respectively. Interestingly, on average, it took 8.9 years for an article to be cited on this topic. USA was the most cited country (33%) followed by 6% for South Africa and the UK. On the other hand, papers published by Swiss researchers scored with highest average citation per paper with 101.1. The top 10 most cited documents on this topic are presented in Table 1 where seven papers were from original research. Our included 7024 studies were published by 23,631 authors from 125 countries (average 0.3 documents per author and 3.4 authors per document), among those, only 0.9% published single-authored documents. Most of the published articles were published in the research fields of (i) Medicine (54.2%) followed by (ii) Immunology and Microbiology (10.7%), (iii) Biochemistry, Genetics and Molecular Biology (10.6%), and (iv) Pharmacology, Toxicology and Pharmaceutics (8.8%).

### 2.3. Trend of Publication and Citation

**Figure 2:**
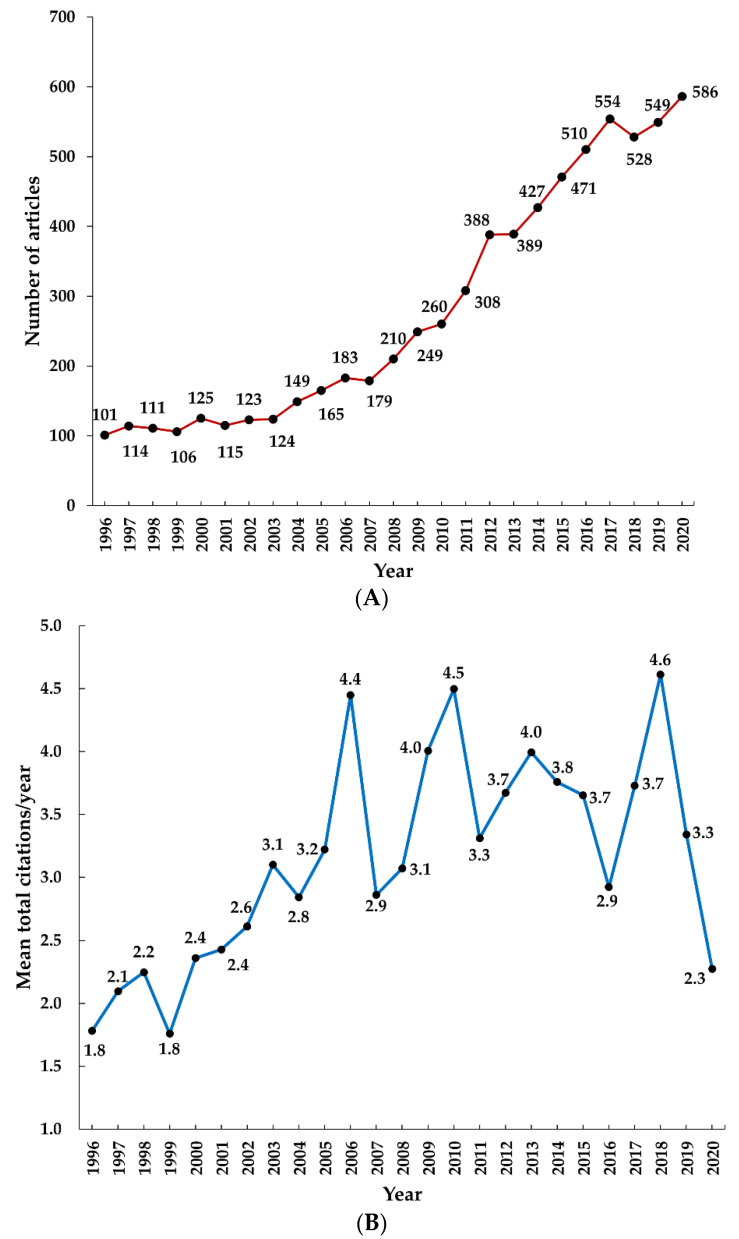
Global annual trend of (**A**) publication and (**B**) citation of antibiotic-resistant active pulmonary tuberculosis research.

In the last 25 years (1996–2020), there has been clear evidence of a persistently increasing publication trend on antibiotic-resistant active pulmonary TB research, with 1996 being the lowest (*n* = 101) and 2020 being the highest (*n* = 586) (Figure 2A). On the other hand, the annual mean citation trend has been increasing slowly, in an inconsistent manner, where the mean citation in 2018 was the maximum with 4.6 (Figure 2B). 

### 2.4. Most Productive Institutions and Their Collaboration Network

**Figure 3:**
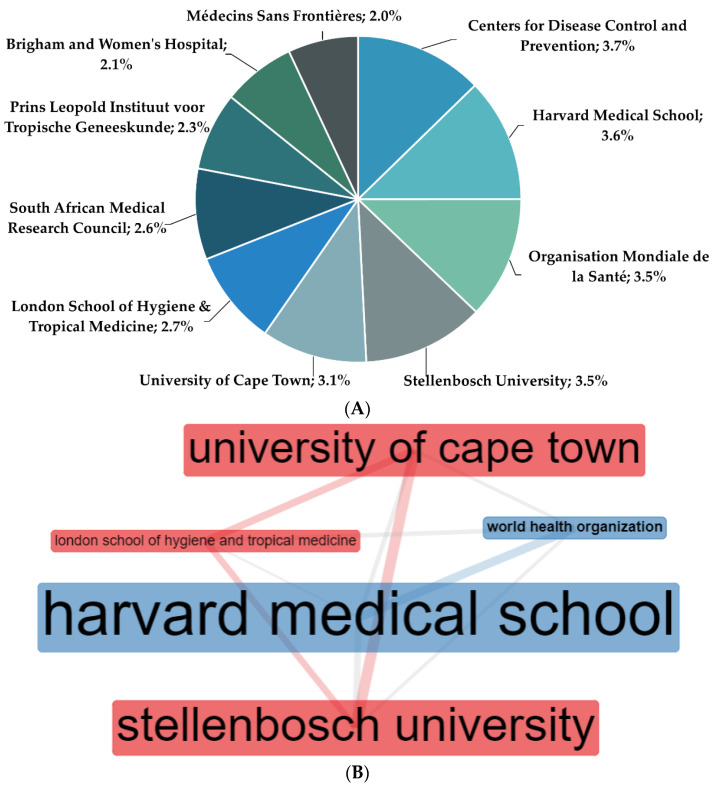
(**A**) Ten most contributing institutions and (**B**) leading five collaboration networks among institutions on antibiotic-resistant active pulmonary tuberculosis research in the past 25 years (1996–2020).

Centers for Disease Control and Prevention was found to be the most productive institution (3.7% papers) followed by Harvard Medical School (3.6% papers) in antibiotic-resistant active pulmonary TB research, worldwide. Based on the leading eigenvalues clustering algorithm assessing betweenness centrality, top five collaborative networks among institutions were identified between (i) Harvard Medical School, (ii) London School of Hygiene and Tropical Medicine, (iii) University of Cape Town, (iv) Stellenbosch University, and (v) World Health Organization (Figure 3).

### 2.5. Most Influential Funding Agencies

**Figure 4:**
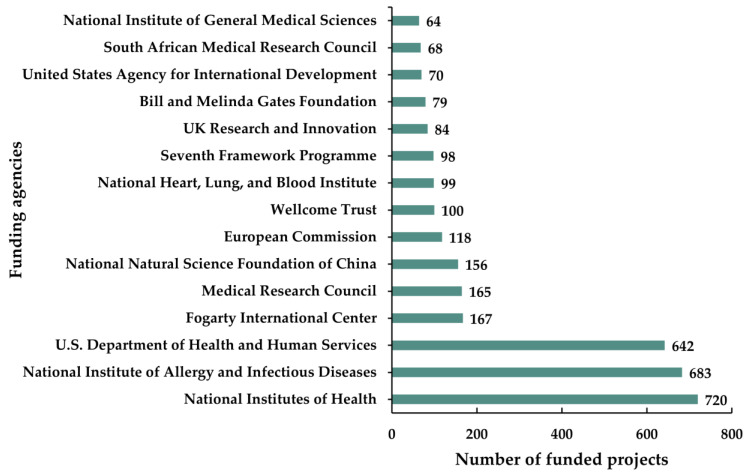
Top 15 funding agencies of antibiotic-resistant active pulmonary tuberculosis research in the past 25 years (1996–2020).

The funding results demonstrate that US agencies dominate as the top funding bodies and (i) National Institutes of Health, (ii) National Institute of Allergy and Infectious Diseases, and (iii) U.S. Department of Health and Human Services were the leading funders, which cumulatively financed in 2045 of the research projects on antibiotic-resistant active pulmonary TB (Figure 4). Besides, other funding agencies are majorly UK based (i.e., Medical Research Council, European Commission, Wellcome Trust, Seventh Framework Programme, UK Research and Innovation), while some are from China (i.e., National Natural Science Foundation of China) and South Africa (i.e., South African Medical Research Council).

### 2.6. Most Contributing Authors and Their Collaboration Networks

**Figure 5:**
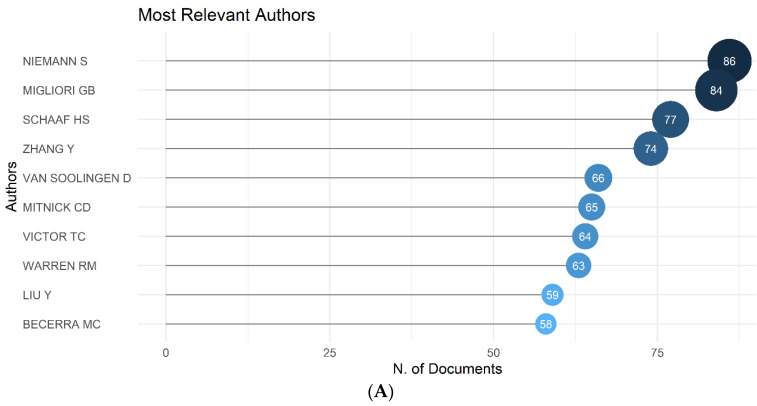

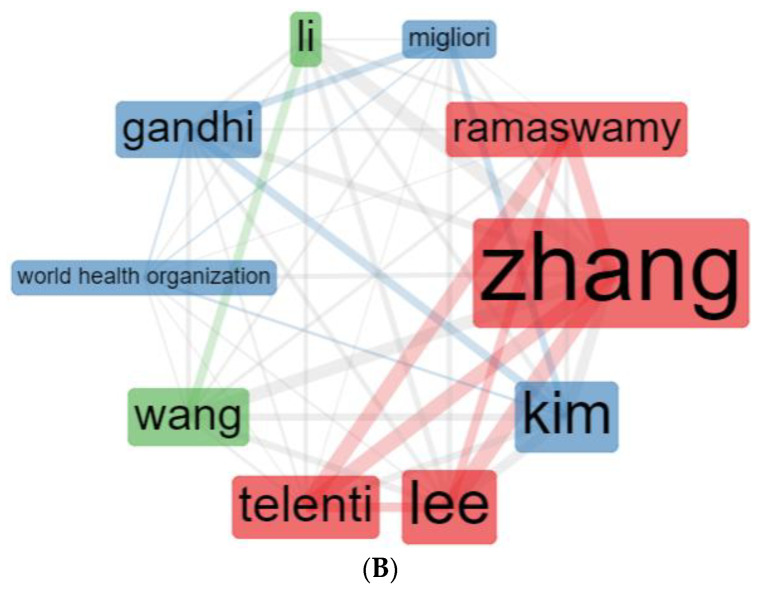
(**A**) Most contributing ten authors and (**B**) co-citation network of authors working on antibiotic-resistant active pulmonary tuberculosis research in the past 25 years (1996–2020).

We found that in the last 25 years on antibiotic-resistant active pulmonary TB topic, in terms of number of documents, (i) Niemann S (*n* = 86), (ii) Migliori GB (*n* = 84) and (iii) Schaaf HS (*n* = 77) were the most contributing authors (Figure 5A). From the co-citation network (Figure 5B), assessing the ‘Leading Eigenvalues’ clustering, based on the high ‘Betweenness’, we observed that the most co-cited authors were (i) Zhang, (ii) Kim, and (iii) Wang.

### 2.7. Most Productive Journals

About 8.8% of the total number of published papers on our topic of interest was published in ‘International Journal of Tuberculosis and Lung Disease’ followed by ‘PLoS One’ (5.1%) and ‘Journal of Clinical Microbiology’ (4.2%) (Table 2). Interestingly, all top ten journals publishing articles on antibiotic-resistant active TB were neither of high impact factor nor Q1/Q2 journals based on the 2020 JCR^®^, WoS. Rather, 60% were in Q1/Q2 category.

### 2.8. World Research Production and Collaborations

**Figure 6:**
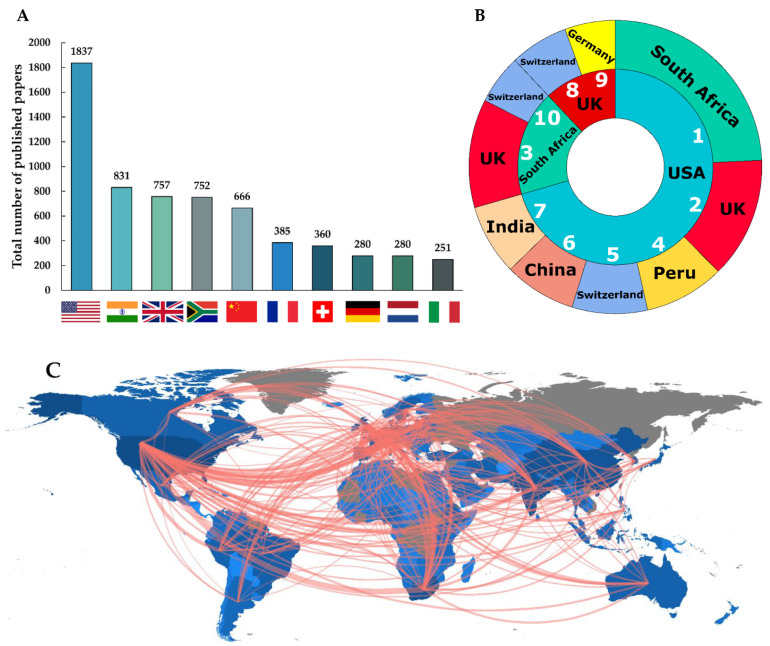
(**A**) Leading publishing countries, (**B**) top ten collaborative countries and (**C**) world collaboration map on antibiotic-resistant pulmonary tuberculosis research (1996–2020).

**Figure 7:**
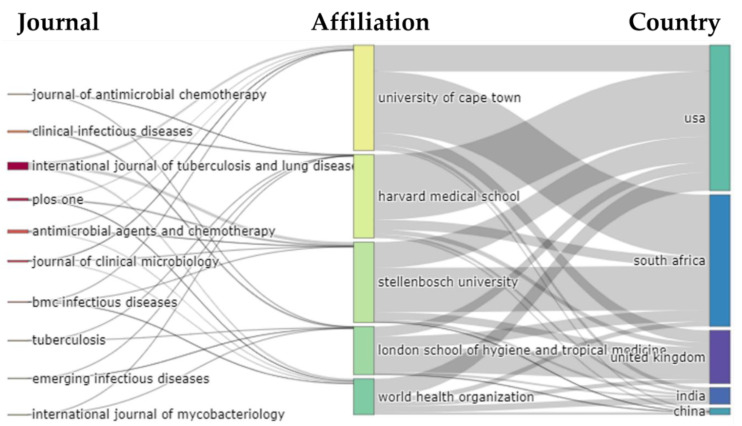
Three-Fields Plot representing the incoming and outgoing flows among top ten journals, affiliations and countries contributing to antibiotic-resistant active pulmonary tuberculosis research in the past 25 years (1996–2020).

Figure 6A represents the top ten countries where the USA, India and the UK were the leading publishing countries on the theme of antibiotic-resistant active pulmonary TB. We found a total of 3014 combinations of country-based collaborations among which we represented the most significant ten collaborations worldwide on this research topic. From the sunburst plot (Figure 6B), we observed that between 1996 and 2020, most of the published collaborative works were between USA → South Africa (*n* = 373); USA → UK (*n* = 205) and South Africa → UK (*n* = 184). Figure 6C represents the worldwide collaboration map of the researchers working on antibiotic-resistant active pulmonary TB. Considering the top ten journals, affiliations, and countries contributing to antibiotic-resistant pulmonary active TB research in the last 25 years (1996–2020), we constructed the three-fields plot which represented the incoming and outgoing flows between these fields where the height of the rectangular nodes in the collaboration network is linked to the frequency of occurrence (Figure 7).

### 2.9. Treemap and Thematic Map

**Figure 8:**
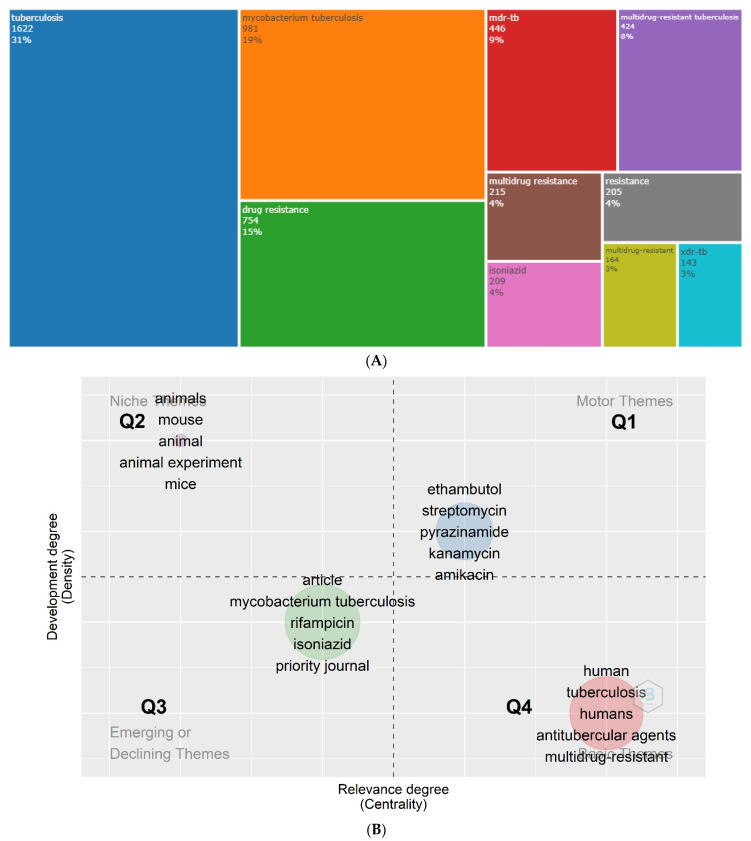
(**A**) TreeMap representing top ten author’s keywords and (**B**) Thematic Map using keywords plus utilised in the research of antibiotic-resistant active pulmonary tuberculosis (1996–2020).

The represented TreeMap signifies the top ten author’s keywords where the top three keywords, (i) tuberculosis (31%), (ii) *Mycobacterium tuberculosis* (19%), and (iii) drug resistance (15%), were used by the authors on this research topic between 1996 and 2020 (Figure 8A). The Thematic Map was constructed using the Keywords Plus (Figure 8B) which are the frequently appearing keywords that exist in the titles of the cited references on the article, however, not found in the title of the paper itself. The Thematic Map was produced based on a two-dimensional matrix that included two kinds of measurements: centrality (X-axis, indicating the importance of a theme) and density (Y-axis, indicating the development of a theme). Based on our Thematic Map, the motor themes at the Q1 included five antibiotics (ethambutol, streptomycin, pyrazinamide, kanamycin and amikacin) representing that these are both important and well-developed themes. Q2 representing the niche themes included animal experiment-associated keywords indicating that these are highly developed however isolated themes. The 3rd quadrant items refer to the emerging or declining themes and the 4th quadrant items indicated the transversal and basic themes, including ‘antitubercular agents’ and ‘multidrug-resistant’. In the Thematic Map, the size of the bubbles is proportionate to the number of times the term appears. The density rank and centrality rank (from high to low) can be expressed as Q2 > Q1 > Q3 > Q4 and Q4 > Q1 > Q3 > Q2, respectively.

## 3. Discussion

The outcomes of various bibliometric analyses have drawn interest from the scientific communities in recent years [21,22]. Bibliometric analysis and scientific mapping are now advancing rapidly. Due to its useful method of evaluating the merits, progress and research trend of a specific topic area by several bibliometric indicators, this tool is used in many different study fields including medical and health sciences, and provides unique idea and vast variety of applications in several study domains making it a crucial research platform [23,24]. Bibliometric analysis can explore the impact of any research field, the impact of a set of researchers, the impact of a particular paper, or help to identify particularly impactful papers understanding the overall intellectual landscape within a specific field of research [22,25].

In our bibliometric analysis on antibiotic-resistant active pulmonary TB research of the past 25 years (1996–2020), we observed that the number of articles steadily increased which is similar to another 10-year bibliometric analysis on overall TB research [10]. The mean citation has been declining in recent years (2019 and 2020), which is most likely due to the fact that older publications are cited more than newly published papers from the same year [26]. Our findings indicate that majority of the publications on this topic were from the USA which is not surprising as the USA spends more on TB research and development than other countries [27]. In addition, 60% of the top ten productive countries are from the Europe, with the UK being at the top of the list, which can again be correlated with the number of research fundings these countries secure. Among the Asian countries, India and China are at the top ten list of productivity on antibiotic-resistant active pulmonary TB research. Previous bibliometric analyses on TB also observed the similar publication and citation trends [10,28]. Among the BRICS countries, although the contribution to overall TB research was high from all of those five countries (i.e., Brazil, Russia, India, China and South Africa) [10], from our analysis, South Africa, China and India were listed in the top ten contributing countries. The highest contribution from these three countries can probably be correlated to the high percentage of allocated global fundings to this group (46% in 2017 46% and 57% in 2020) and due to underreporting of TB research from Russia and Brazil [1]. Although Indian and Chinese funding agencies were noted in the previous analysis on overall TB research’s funding list (especially in epidemiological and fundamental research projects) [10], in our antibiotic-resistant TB research-oriented analyses, these funding bodies were not notable in the top list. Therefore, both India and China can actively allocate their TB fundings in antibiotic-resistant research of TB as antibiotic-resistance patterns against TB have high burdens in these countries [29,30]. Consequently, the global progress towards the elimination of TB will need the support of BRICS nations, to optimise the strategies and to ensure that research outcomes also become effective in policymaking.

When looking at the top journals based on the number of papers published, it is worth noting that not all of them have a high impact factor, and one of the top ten (i.e., International Journal of Mycobacteriology) wasn’t even indexed in Science Citation Index Expanded (SCIE), WoS based on the 2020 JCR^®^, but rather in Emerging Sources Citation Index, WoS based on the 2020 JCR^®^ (without impact factor). Although this India-based journal is not indexed in SCIE, WoS, India remains as one of the topmost (ranked 6th) cited countries according to our analysis. Therefore, we agree that the impact factor of paper publications does not necessarily represent the quality of the papers [31,32].

When we looked at the top 100 collaborations among different countries from Africa, South America and Asia with rest of the world. We found only three African (Tanzania, Uganda and Ethiopia), two South American (Brazil and Peru), and four Asian countries (Thailand, Philippines, Pakistan and Hong Kong). Interestingly, when we investigated top 100 countries with the most citations within these three continents, surprisingly, in that top list, we noticed 26 African countries (Algeria, Botswana, Burkina Faso, Congo, Egypt, Ethiopia, Gambia, Ghana, Guinea, Cameroon, Central African Republic, Kenya, Lesotho, Madagascar, Malawi, Mali, Morocco, Mozambique, Niger, Nigeria, South Africa, Tanzania, Tunisia, Uganda, Zambia, and Zimbabwe), six countries from South America (Argentina, Colombia, Ecuador, Peru, Trinidad and Tobago and Venezuela) and 24 countries from Asia (Bangladesh, Cambodia, Hong Kong, India, Indonesia, Iran, Iraq, Japan, Kazakhstan, Korea, Kuwait, Lebanon, Malaysia, Mongolia, Myanmar, Nepal, Pakistan, Philippines, Saudi Arabia, Singapore, Sri Lanka, Thailand, Uzbekistan, Vietnam). This indicates that even though researchers from these African, South American, and Asian countries are producing quality papers (based on their worldwide citations on this topic) on antibiotic-resistant active pulmonary TB, their worldwide collaboration is in some way limited, except South Africa, Peru, India, and China. Our observation is with an agreement with an early PubMed-based 10-year (1997–2006) bibliometric analysis on overall TB by Ramos et al. [28], who revealed that the TB research effort was higher in low-income countries than in high-income countries. Hence, researchers from high-income countries should enhance their worldwide collaborations with countries in the African, South American and Asian regions—which shows a good track record of quality papers and at the same time faces high antibiotic-resistance burden of TB [33,34,35]. The findings from this collaboration will eventually help us win the global fight against antibiotic-resistant TB. Likewise, the journals managed by the non-English countries should not only improve their local research databases, but also get indexed into international databases, such as Scopus, PubMed, and WoS, so that their findings on antibiotic-resistant TB are easily disseminated and based on the real scenario of TB burden. Researchers, funding bodies, and policymakers can develop appropriate strategies to fight against TB antibiotic-resistance.

### Strengths and Limitations

There are some notable strengths. This is the first bibliometric analysis analysing the research scenario on antibiotic-resistant active pulmonary TB of 25 years at a global scale. We comprehensively analysed, presented and interpreted the meta-data in an understandable way using one of the most acceptable and appreciated software platforms. We considered articles published in only English language and only 140 non-English papers were excluded (Mainly Spanish, Portuguese, French, Turkish, Russian, German, Polish and Italian), which is not a significant number compared to the total English language publications. Therefore, we presume that excluding those non-English papers did not have any significant quantitative influence in changing the current findings of this bibliometric analysis. 

Nevertheless, there are some limitations. The institutional or author ranks were made by the number of contributions, therefore, these ranks do not represent the quality or impact of the research. For the funded projects, as there were no ways to identify the amount of fundings, a different trend might be visualised if we could retrieve the size of fundings. Although we used total citations and citations per document per year to evaluate study impact or quality, there is criticism that citations may not reflect research impact or quality [31]. As our analysis is entirely based on Scopus database, the outcomes may be biased towards publications in other databases. For example, the non-English speaking countries may have their own indexing databases. As a result, their authors may write in their national language and subsequently publish in their local journals, which are not indexed in Scopus or other internationally recognised databases. This will translate into publication bias where there is a lack of representation of the actual publications on antibiotic-resistant active pulmonary TB in other bibliometric analyses [10,28].

## 4. Methods

### 4.1. Eligibility Criteria and Data Source

In this bibliometric analysis, studies on antibiotic-resistant active pulmonary TB published between 1996 and 2020 as an original article, a review or a conference paper in English language were considered eligible. Scopus database was used, and all the metadata were downloaded in the BibTex format.

### 4.2. Search Strategy

In the advanced search option of Scopus database, using an appropriate combination of Boolean and wildcard search operators, the following keywords were searched: “tuberculosis”, “TB” “resistan*” “susceptibl*” “sensitivit*”. The search was performed on 10 September 2021 and the entire search strategy is presented in Table S1.

### 4.3. Bibliometric Analyses

Data management and bibliometric analyses were conducted using the ***Bibliometrix*** package (version 3.1.4) and ***Biblioshiny*** web apps under the R language (version 4.0.4) inside RStudio integrated development environment software (RStudio, Inc., Boston, MA, USA) (version 1.4.1106) [36,37]. All the main information and characteristics of the included studies were retrieved. The publication and citation trends were constructed throughout a 25-year period. We identified the most productive institutions based on the highest number of paper contributions to the topic between 1996 and 2020 and constructed a collaboration network among the top five institutions using the leading eigenvalues clustering algorithm where the data of 7024 papers were normalized based on the association parameter. The most influential funding agencies financing research on antibiotic-resistant active pulmonary TB in the last 25 years were also retrieved and presented as a clustered bar. We identified the most contributing authors based on the highest number of paper contributions as well as top ten co-citation networks of the influential authors using the leading eigenvalues clustering algorithm with the repulsion force of 0.1. The top ten most cited documents and most productive journals were also identified and some features such as the country of origin, the number of papers published on the topic, h-index, total citations, Journal citation report (JCR^®^), Web of Science (WoS) 2020 Impact factor, journal category and quartile the journal is enlisted in were retrieved. To observe the incoming and outgoing flows among top ten journals, affiliations and countries contributing to antibiotic-resistant pulmonary active TB research in the last 25 years, a three-fields plot was constructed. The world research collaboration was represented by the Collaboration Worldmap where minimum edges were set to ten. A Treemap was prepared to present the top ten author’s keywords used in the included published papers on the topic between 1996 and 2020. Finally, the ‘Thematic Map’ was developed based on four themes, including (i) the motor themes, (ii) the niche themes, (iii) emerging or declining themes, and (iv) the transversal and basic themes. The map was constructed using 1000 Keywords Plus used maximum times by the researchers on the topic in the past 25 years where a minimum of 10 cluster frequencies per thousand documents was placed.

## 5. Conclusions

This bibliometric analysis presents qualitative and quantitative evaluations of the top countries, authors, institutions, leading journals, funding agencies, and global collaboration scenarios of antibiotic-resistant active pulmonary TB. In most areas of antibiotic-resistant TB research, researchers and funding from the USA dominated, followed by the UK. However, researchers from low- and middle-income countries (especially from Africa, South America, and Asia) with high research productivity and TB burden could collaborate with researchers from high-income countries with low TB burden to produce more productive and translational antibiotic-resistant Tb research.

## Figures and Tables

**Table 1:** The top 10 most cited documents on antibiotic-resistant active pulmonary tuberculosis research (1996–2020).

Rank	Study ID [references]	Title of the Document	Document Type	Journal Name	Total Citations	DOI
1	Boehme 2010 [11]	Rapid Molecular Detection of Tuberculosis and Rifampin Resistance	Research Article	The New England Journal of Medicine	1609	10.1056/NEJMoa0907847
2	Gandhi 2006 [12]	Extensively drug-resistant tuberculosis as a cause of death in patients co-infected with tuberculosis and HIV in a rural area of South Africa	Research Article	The Lancet	1307	10.1016/S0140-6736(06)69573-1
3	Tacconelli 2018 [13]	Discovery, research, and development of new antibiotics: the WHO priority list of antibiotic-resistant bacteria and tuberculosis	Research Article	The Lancet Infectious Diseases	1259	10.1016/S1473-3099(17)30753-3
4	Ramaswamy 1998 [14]	Molecular genetic basis of antimicrobial agent resistance in Mycobacterium tuberculosis: 1998 update	Review article	Tubercle and Lung Disease	886	10.1054/tuld.1998.0002
5	Palomino 2002 [15]	Resazurin Microtiter Assay Plate: Simple and Inexpensive Method for Detection of Drug Resistance in *Mycobacterium tuberculosis*	Research Article	Antimicrobial Agents and Chemotherapy	790	10.1128/AAC.46.8.2720-2722.2002
6	Gandhi 2010 [16]	Multidrug-resistant and extensively drug-resistant tuberculosis: a threat to global control of tuberculosis	Review article	The Lancet	751	10.1016/S0140-6736(10)60410-2
7	Boehme 2011 [17]	Feasibility, diagnostic accuracy, and effectiveness of decentralised use of the Xpert MTB/RIF test for diagnosis of tuberculosis and multidrug resistance: a multicentre implementation study	Research Article	The Lancet	743	10.1016/S0140-6736(11)60438-8
8	Centers for Disease Control and Prevention (CDC) 2006 [18]	Emergence of *Mycobacterium tuberculosis* with extensive resistance to second-line drugs—Worldwide, 2000–2004	Review article	Morbidity and Mortality Weekly Report	681	Not available
9	Diacon 2009 [19]	The Diarylquinoline TMC207 for Multidrug-Resistant Tuberculosis	Research Article	The New England Journal of Medicine	642	10.1056/NEJMoa0808427
10	Helb 2010 [20]	Rapid Detection of *Mycobacterium tuberculosis* and Rifampin Resistance by Use of On-Demand, Near-Patient Technology	Research Article	Journal of Clinical Microbiology	625	10.1128/JCM.01463-09

**Table 2:** Top ten journals publishing papers on antibiotic-resistant active pulmonary tuberculosis research (1996–2020).

Rank	Journal Name	Country	Number of Papers Published on the Topic (%), *n* = 7024	h-Index	Total Citations	JCR^®^ 2020 Impact Factor	JCR^®^ 2020 Category (Quartile)
1	International Journal of Tuberculosis and Lung Disease	France	616 (8.8%)	59	16183	2.373	Respiratory system (Q4); Infectious diseases (Q4)
2	PLoS One	USA	358 (5.1%)	46	8561	3.240	Multidisciplinary sciences (Q2)
3	Journal of Clinical Microbiology	USA	297 (4.2%)	68	14,535	5.948	Microbiology (Q1)
4	Antimicrobial Agents and Chemotherapy	USA	288 (4.1%)	69	15,916	5.191	Pharmacology & Pharmacy (Q1); Microbiology (Q2)
5	BMC Infectious Diseases	England	157 (2.2%)	26	2672	3.090	Infectious Diseases (Q3)
6	Clinical Infectious Diseases	USA	146 (2.1%)	49	6700	9.079	Microbiology (Q1); Infectious Diseases (Q1); Immunology (Q1)
7	Tuberculosis	England	144 (2.1%)	28	2619	3.131	Microbiology (Q3); Respiratory System (Q3); Immunology (Q3)
8	Journal of Antimicrobial Chemotherapy	England	112 (1.6%)	44	5198	5.790	Microbiology (Q1); Pharmacology & Pharmacy (Q1); Infectious Diseases (Q1)
9	International Journal of Mycobacteriology	India	107 (1.5%)	13	612	ESCI	Microbiology (Q4) *; Infectious Diseases (Q4)
10	Emerging Infectious Diseases	USA	100 (1.4%)	32	3804	6.883	Infectious Diseases (Q1); Immunology (Q1)

JCR: journal citation reports, * Based on journal citation indicator.

## Data Availability

All data generated or analysed during this study are included in this published article and Supplementary Materials.

## Supplementary Materials

The following supporting information can be downloaded at: www.mdpi.com/article/10.3390/antibiotics11081012/s1, Table S1: Search strategy.
