# Supplementary material for: A Global Bibliometric Analysis on Antibiotic-Resistant Active Pulmonary Tuberculosis over the Last 25 Years (1996–2020)
Source: Antibiotics (Basel). 2022 Jul 27;11(8):1012. doi: 10.3390/antibiotics11081012 (PMC9405510; doi:10.3390/antibiotics11081012)
Supplement: Supplementary file 1 [file antibiotics-11-01012-s001.zip › antibiotics-1826902-supplementary.pdf]

Table S1. Search strategy

**Scopus**	TITLE(tuberculosis OR TB) AND TITLE(resistan* OR susceptibl* OR sensitivit*) AND NOT TITLE(nontuberculosis OR non-tuberculosis OR latent OR extrapulmonary) AND (LIMIT-TO (SRCTYPE,"j") OR LIMIT-TO (SRCTYPE,"p")) AND (LIMIT-TO (DOCTYPE,"ar") OR LIMIT-TO (DOCTYPE,"re") OR LIMIT-TO (DOCTYPE,"cp")) AND (LIMIT-TO (LANGUAGE,"English")) AND (LIMIT-TO (PUBYEAR,2020) OR LIMIT-TO (PUBYEAR,2019) OR LIMIT-TO (PUBYEAR,2018) OR LIMIT-TO (PUBYEAR,2017) OR LIMIT-TO (PUBYEAR,2016) OR LIMIT-TO (PUBYEAR,2015) OR LIMIT-TO (PUBYEAR,2014) OR LIMIT-TO (PUBYEAR,2013) OR LIMIT-TO (PUBYEAR,2012) OR LIMIT-TO (PUBYEAR,2011) OR LIMIT-TO (PUBYEAR,2010) OR LIMIT-TO (PUBYEAR,2009) OR LIMIT-TO (PUBYEAR,2008) OR LIMIT-TO (PUBYEAR,2007) OR LIMIT-TO (PUBYEAR,2006) OR LIMIT-TO (PUBYEAR,2005) OR LIMIT-TO (PUBYEAR,2004) OR LIMIT-TO (PUBYEAR,2003) OR LIMIT-TO (PUBYEAR,2002) OR LIMIT-TO (PUBYEAR,2001) OR LIMIT-TO (PUBYEAR,2000) OR LIMIT-TO (PUBYEAR,1999) OR LIMIT-TO (PUBYEAR,1998) OR LIMIT-TO (PUBYEAR,1997) OR LIMIT-TO (PUBYEAR,1996))
